# Exploration of *Plasmodium vivax* transmission dynamics and recurrent infections in the Peruvian Amazon using whole genome sequencing

**DOI:** 10.1186/s13073-018-0563-0

**Published:** 2018-07-04

**Authors:** Annie N. Cowell, Hugo O. Valdivia, Danett K. Bishop, Elizabeth A. Winzeler

**Affiliations:** 1 Division of Infectious Diseases, Department of Medicine, University of California, San Diego, 9500 Gilman Dr., La Jolla, CA 92093 USA; 2 U.S. Naval Medical Research No. 6, Venezuela Ave, Block 36, Bellavista, Callao, Peru; 3 Division of Host-Microbe Systems & Therapeutics, Department of Pediatrics, UC San Diego, 9500 Gilman Dr., La Jolla, CA 92093 USA

**Keywords:** Malaria, *Plasmodium vivax*, Relapse, Primaquine, Whole genome sequencing, Population genetics

## Abstract

**Background:**

*Plasmodium vivax* poses a significant challenge to malaria elimination due to its ability to cause relapsed infections from reactivation of dormant liver parasites called hypnozoites. We analyzed 69 *P*. *vivax* whole genome sequences obtained from subjects residing in three different villages along the Peruvian Amazon. This included 23 paired *P*. *vivax* samples from subjects who experienced recurrent *P*. *vivax* parasitemia following observed treatment with chloroquine and primaquine.

**Methods:**

Genomic DNA was extracted from whole blood samples collected from subjects. *P*. *vivax* DNA was enriched using selective whole genome amplification and whole genome sequencing. We used single nucleotide polymorphisms (SNPs) from the core *P*. *vivax* genome to determine characteristics of the parasite population using discriminant analysis of principal components, maximum likelihood estimation of individual ancestries, and phylogenetic analysis. We estimated the relatedness of the paired samples by calculating the number of segregating sites and using a hidden Markov model approach to estimate identity by descent.

**Results:**

We present a comprehensive dataset of population genetics of *Plasmodium vivax* in the Peruvian Amazonian. We define the parasite population structure in this region and demonstrate a novel method for distinguishing homologous relapses from reinfections or heterologous relapses with improved accuracy. The parasite population in this area was quite diverse with an estimated five subpopulations and evidence of a highly heterogeneous ancestry of some of the isolates, similar to previous analyses of *P*. *vivax* in this region. Pairwise comparison of recurrent infections determined that there were 12 homologous relapses and 3 likely heterologous relapses with highly related parasites. To the best of our knowledge, this is the first large-scale study to evaluate recurrent *P*. *vivax* infections using whole genome sequencing.

**Conclusions:**

Whole genome sequencing is a high-resolution tool that can identify *P*. *vivax* homologous relapses with increased sensitivity, while also providing data about drug resistance and parasite population genetics. This information is important for evaluating the efficacy of known and novel antirelapse medications in endemic areas and thus advancing the campaign to eliminate malaria.

**Electronic supplementary material:**

The online version of this article (10.1186/s13073-018-0563-0) contains supplementary material, which is available to authorized users.

## Background

Malaria is a tropical disease caused by *Plasmodium* parasites which remains one of the most important public health problems worldwide [1]. The disease is endemic in more than 90 countries and poses a risk to nearly 2.5 billion people with an estimated 212 million cases and 429,000 deaths in 2015 [1]. Of the five species known to infect humans, *P*. *falciparum* and *P*. *vivax* stand out as the leading causes of malaria in endemic areas. Although *P*. *vivax* infections are not as deadly as those caused by *P*. *falciparum*, it is the most geographically widespread malaria species leading to enormous morbidity and severe disease [2, 3]. Implementation of malaria control strategies has significantly reduced malaria incidence and deaths between 2000 and 2015 [4]. This rapid reduction has been especially important for the control of *P*. *falciparum* in several endemic regions in the Americas; however, *P*. *vivax* has now replaced *P*. *falciparum* as the predominant species outside Africa [1]. The reasons for this change are related to the unique biological features of *P*. *vivax* including (i) the higher potential of transmission and infectivity to other species of mosquitoes compared to other *Plasmodium* species [5] and (ii) the ability to generate long lasting dormant hepatic parasites (hypnozoites) that can become active weeks, months, or years after the first infection, resulting in relapse [6].

Relapses represent a major threat to malaria elimination worldwide because hypnozoites are undetectable by current diagnostic tests [2] and present a new opportunity for malaria transmission once activated. Conventional medications used to treat blood-stage infections such as chloroquine are not efficacious against hypnozoites. Currently, the only treatment licensed to prevent *Plasmodium vivax* relapses through killing hypnozoites is primaquine, which frequently causes gastrointestinal side effects, poses a risk of hemolysis in people with G6PD deficiency [2, 7], and has decreased efficacy in people with mutations in the *cyp2d6* gene that encodes cytochrome P450 2D6 [8]. Furthermore, measuring primaquine efficacy is challenging in many endemic sites because administration is not adequately supervised. Additionally, because relapses can be due to activation of hyponozoites from the most recent infection (homologous relapse) or activation of hypnozoites from prior infections (heterologous relapse), it remains difficult to distinguish whether recurrent parasitemia is due to relapse or reinfection from a new mosquito bite.

Whole genome sequencing (WGS) can enable highly detailed comparisons of recurrent *P*. *vivax* infections [9] and thus can identify homologous relapses with greater accuracy. It also provides further information about parasite population structure, polymorphisms in drug resistance markers, and genomic regions under selection [10, 11]. Previous methods used to distinguish homologous relapses from reinfections include microsatellite marker comparison [12, 13] and deep sequencing of hypervariable genes such as merozoite surface protein 1 (*msp1*) [14]. However, these methods have a limited resolution that could affect an accurate differentiation of recurrent infections. For example, a prior study of a traveler who returned to a malaria non-endemic region compared *P*. *vivax* whole genome sequences from subsequent episodes of recurrent parasitemia and demonstrated that relapses could occur with a meiotic sibling, or closely related *P*. *vivax* parasite strain, likely representing recombination that had occurred in the mosquito midgut at the time of the initial infection [9]. In this case, using microsatellite markers alone to compare infections would have mistakenly determined that the recurrent infection was due to reinfection rather than relapse.

**Fig. 1:**
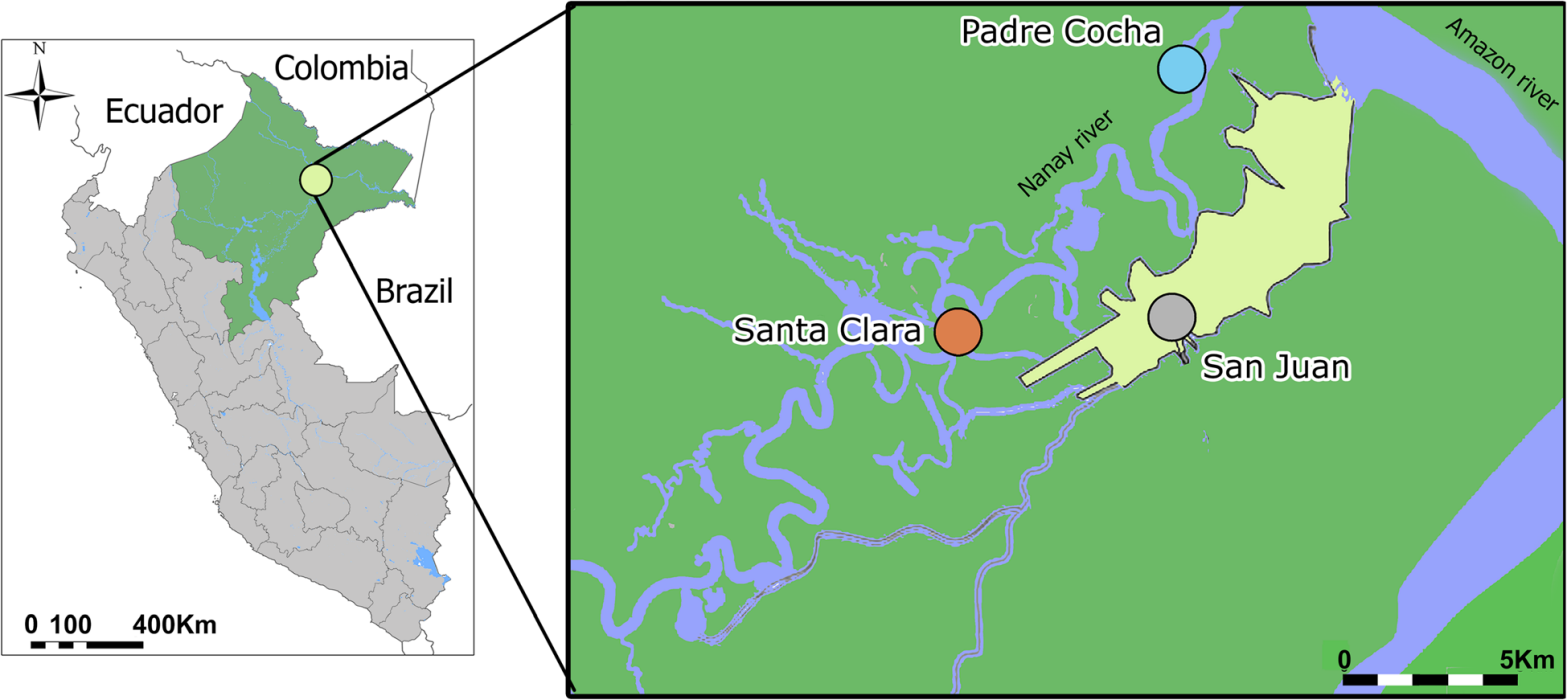
Map of the study area. This figure illustrates the Peruvian region of Loreto and its capital city of Iquitos (colored in yellow). Samples were collected from three villages located in the near proximity to the city. This is an original figure made using ArcGIS version 10.0

Here, we analyze 69 *P*. *vivax* whole genome sequences obtained from 46 subjects living in three villages near the city of Iquitos in the Peruvian Amazon region of Loreto (Fig. 1). This genomic set includes 46 *P*. *vivax* sequences that were derived from 23 paired samples obtained from the same subject collected before treatment with primaquine and chloroquine and at the time of recurrent *P*. *vivax* parasitemia following treatment. Genomic data was used to assess gene diversity, population structure, and drug resistance patterns within the population. In addition, we compared the 23 *P*. *vivax* paired samples to assess whether they represented homologous relapses or were more likely to be reinfections or heterologous relapses.

## Methods

### Subject sample collection and preparation

Whole blood samples were collected from subjects with symptomatic *P*. *vivax* infections from the endemic region of Iquitos in the northeastern Peruvian Amazon during a previous study conducted by the US Naval Medical Research Unit 6 (NAMRU-6) between 2006 and 2008 to evaluate three different primaquine regimens: 0.5 mg/kg × 5 days, 0.5 mg/kg × 7 days, and 0.25 mg/kg × 14 days [15]. This included 23 pairs of samples collected from subjects at two time points: initial infection prior to treatment with chloroquine and primaquine, and recurrent parasitemia between 36 to 210 days after observed treatment (Additional file 1: Table S1). Since one goal of the current study was to identify genetic markers or primaquine resistance, subjects with recurrent parasitemia between 17 and 35 days following treatment were considered to have potential chloroquine resistance and were excluded from this analysis [16]. Most subjects who received the shortest regimen (0.5 mg/kg × 5 days) were excluded from the present analysis since they had statistically higher rates of relapse compared to the other regimens [15]. Thick blood smears were examined to identify the parasite species and to determine the level of parasitemia. Parasite density was calculated by counting the number of asexual parasites per 200 white blood cells in the thick smear (assuming an average of 6000 white blood cells per μl). Two microscopists examined each blood smear independently, and a third microscopist gave confirmation in the event of a discrepancy. The final parasite density was calculated as the average of density readings from the two concordant microscopists. Microsatellite genotyping was performed using six neutral microsatellite markers as previously described [15]. Whole blood samples were collected in the field using EDTA-containing vacutainer tubes, and samples were frozen and transported to the central laboratory for further processing.

### Selective whole genome amplification (SWGA)

DNA was isolated from thawed whole blood using QIAamp DNA Blood Mini Kit (Qiagen) following the manufacturer’s recommendation and as described elsewhere [17]. Samples were subsequently resuspended in TE buffer, and genomic DNA was quantified using a Qubit 2.0 fluorometer (ThermoFisher). Thirty to 70 ng of input DNA was added to a 50-μl reaction containing 3.5 μM SWGA primers, 30 U phi29 DNA polymerase enzyme (New England Biolabs), phi29 DNA buffer (New England Biolabs), 1% bovine serum albumin, and water as previously described [18, 19]. The primer set used consists of 12 primers: 5′-AACGAAGC*G*A-3′, 5′-ACGAAGCG*A*A-3′, 5′-ACGACGA*A*G-3′, 5′-ACGCGCA*A*C-3′, 5′-CAACGCG*G*T-3′, 5′-GACGAAA*C*G-3′, 5′-GCGAAAAA*G*G-3′, 5′-GCGAAGC*G*A-3′, 5′-GCGGAAC*G*A-3′, 5′-GCGTCGA*A*G-3′, 5′-GGTTAGCG*G*C-3′, and AACGAAT*C*G. The reaction was carried out on a thermocycler and consisted of a ramp down from 35 to 30 °C (10 min per degree), 16 h at 30 °C, 10 min at 65 °C, and hold at 4 °C. The samples were diluted 1:1 with DNAse-free and RNAse-free water and purified with Ampure XP beads (Beckman-Coulter) at a 1:1 ratio per the manufacturer’s protocol.

### Whole genome sequencing

Next-generation sequencing libraries of SWGA products were prepared using the Nextera XT DNA preparation kit (Illumina) per the manufacturer’s protocol. These samples were pooled and clustered on a Hiseq 2500 (Illumina) in Rapid Run mode with 100 base pair paired end reads. Raw fastq files were aligned to the Sal-1 reference genome (PlasmoDB version 13, http://plasmodb.org/common/downloads/release-13.0/PvivaxSal1/fasta/data/) using the Burroughs-Wheeler Aligner (version 0.7.8) [20] and samtools (version 0.1.19) [21, 22] as previously described in the Platypus pipeline [23]. Picard (version 2.0.1) was used to remove unmapped reads, and the Genome Analysis Toolkit (GATK) [24] was used to realign the sequences around the indels.

### Variant calling and analysis

We followed the GATK’s best practices to call variants [25, 26]. The aligned sequences were run through GATK’s HaplotypeCaller in “reference confidence” mode to create genomic GVCF files for each sample. This reference confidence model highlights areas of the genome that are likely to have variation and produces a comprehensive record of genotype likelihoods and annotations for each site. The samples were joint genotyped using the GenotypeGVCFs tool. Variants were further filtered based on quality scores and sequencing bias statistics based on default parameters from GATK. SNPs were filtered out if they met any of the following criteria: quality depth (QD) < 2.0, mapping quality (MQ) < 50.0, phred-scaled *p* value using Fisher’s exact test to detect strand bias (FS) > 60.0, symmetric odds ratio (SOR) > 4.0, *Z*-score from Wilcoxon rank sum test of alternative vs. reference read mapping qualities (MQRankSum) < − 12.5, and ReadPosRankSum (RPRS) < − 8.0. Variants were annotated using snpeff (version 4.2) [27]. SNP density was visualized in R for the detection of highly polymorphic regions. The core *P*. *vivax* genome, as defined by Pearson et al. [11], was used for further genome analysis.

*F*_ws_ of samples with the highest genome coverage was estimated using *moimix* (https://github.com/bahlolab/moimix), a package available through R. The package calculates *F*_ws_ statistic using the equation *F*_ws_ = 1 − (*Hw/Hs*), where *Hw* is the within-host heterozygosity and *Hs* is the population-level heterozygosity [28, 29].

### Population structure analysis

The core *P*. *vivax* genome resulting from GATK was used for discriminant analysis of principal components using the adegenet package implemented in R [30]. DAPC constitutes a powerful tool for exploring the population structure without relying on a defined genetic model, Hardy-Weinberg equilibrium or linkage disequilibrium.

In order to provide an additional estimation of the parasite subpopulation in these foci, we performed maximum likelihood estimation of individual ancestries using ADMIXTURE [31]. This tool identifies the probability of membership of each individual to a cluster. We tested multiple runs by imputing successive values of *K* from 1 to 8 under a tenfold cross-validation procedure with 2000 pseudoreplicates under different initial seed values for each *K* and used ADMIXTURE’s cross-validation to identify the most likely value of *K*. The pophelper R package and the software CLUMPP [32] were used to obtain the optimal alignments of the replicates for each *K* value and to make multiline plots.

### Phylogenetic analysis

The core *P*. *vivax* genome was used to assess the phylogenetic relationship of the isolates collected in the Iquitos region. For this purpose, SNPs were used to generate genomic sequences for each isolate in GATK. Genomic sequences were subsequently aligned with MAFFT, and the resulting multiple sequence alignment was analyzed on jModelTest2 [33] for statistical selection of the best-fit models according to the Akaike information criterion (AIC), decision theory method (DT), and Bayesian information criterion (BIC). A phylogenetic analysis was performed under a maximum likelihood approach on RAxML [34] using the general time reversible model selected by jModelTest with 1000 pseudoreplicates. Figtree v.1.4.24 was used to generate and visualize the resulting maximum likelihood tree.

### Paired sample comparisons

We employed BioPerl to estimate the number of segregating sites between the baseline and post-treatment sample of each potential relapse and across all the permutated sample pairs in the population. In order to identify homologous relapses in the sampled population, we compared the number of segregating sites between the relapse pairs versus the average number of segregating sites on the permutated pairs minus 1.5 standard deviations. Potential relapse pairs were also screened under a hidden Markov model approach implemented in the glpsnort pipeline to detect genomic segments that could be identical by descent [35, 36]. In addition, we performed a direct pairwise comparison on filtered SNP data using custom scripts. SNPs were considered the same if they had the same read call. If the call at a locus was heterozygous for either samples, they were considered the same if ≥ 80% of reads at that locus for each sample were the same read call. Matlab was used to generate the comparison plots across the chromosomes. Polymorphisms of the cytochrome P450 2D6 gene in the homologous relapses were identified using the xTAG CYP2D6 kit (Luminex, USA) on a Luminex platform.

## Results

### Sample collection and whole genome sequencing

The samples used in this study were collected during a clinical trial performed in three villages surrounding Iquitos, Peru, to assess the efficacy of three different primaquine regimens [15]. We obtained 69 high-quality *P*. *vivax* whole genome sequences directly from subject samples by selective whole genome amplification (SWGA) performed on genomic DNA (gDNA) extracted from whole blood samples [19]. We aligned these sequences to the *P*. *vivax* Salvador-1 reference genome and obtained an average of 24X coverage with 61.1% ± 23.5 of the genome covered by ≥ 5 reads (Additional file 2: Table S2). We identified a total of 24,571 high-quality single nucleotide polymorphisms (SNPs) in the core genome of this group of these sequences (Additional file 3: Table S3).

### Chromosome SNP density

We investigated the density of SNPs in the 69 sequences at the chromosome level to identify regions of increased variability in the core genome. We identified regions with high SNP density on chromosomes 3, 6, 10, and 13. Genes located in these highly variable regions include virulence factors such as members of the plasmodium interspersed repeat (*pir*) gene family (Chr 03) that mediate immune evasion and parasite host interaction [37], merozoite surface protein 8 (*msp8*) (Chr10) which is a potential *P*. *vivax* vaccine candidate [38], variant interspersed repeat 21 (*vir21*) (Chr 13) which participates in evasion via transcriptional switching [39], and several hypothetical proteins (PVX_110960, PVX_110955, PVX_110950, PVX_110945, PVX_110940, and PVX_110945) located on a SNP dense region on chromosome 6 (Additional file 4: Figure S1).

### Diversity of drug-resistant gene orthologs

Our understanding of the genetic changes that impart phenotypic drug resistance in *P*. *vivax* is significantly limited compared to *P*. *falciparum* mainly due to increased challenges with in vitro culture of the parasite and lack of validated drug susceptibility assays. Thus, drug resistance genes in *P*. *vivax* such as *pvmdr1* were previously identified based on orthologous genes in *P*. *falciparum*. In our sample set, we detected several SNPs in orthologs of known drug resistance genes in *P*. *falciparum* comprising up to 47 different haplotypes, consistent with prior whole genome sequencing studies of *P*. *vivax* from this region [40] (Table 1).

**Table 1:** Homozygous single nucleotide polymorphisms (SNPs) in *P*. *falciparum* drug resistance gene orthologs detected in the 69 *Plasmodium vivax* sequences

Locus	Chr	Position	Ref	Alt	Amino acid	Samples*
*pvcrt-0* (PVX_087980)	1	331,151	T	C	Intron	61 (67)
		331,819	G	A	Intron	29 (69)
		332,453	T	C	Intron	59 (68)
		332,874	A	C	Intron	67 (68)
*pvdhfr* (PVX_089950)	5	964,760	C	G	Phe57Leu	2 (66)
		964,762	G	A	Ser58Asn	4 (63)
		964,763	C	G,A	Ser58Arg	55 (59)
		964,939	G	A	Ser117Asn	58 (62)
*pvmdr1* (PVX_080100)	10	362,888	A	G	Phe1070Leu	6 (49)
		363,223	A	G	Thr958Met	44 (46)
		363,374	T	G	Met908Leu	46 (47)
		365,435	C	A	Val221Leu	9 (49)
Multidrug resistance protein 2 (PVX_118100)	12	2,413,549	A	T	Tyr514Phe	6 (58)
		2,415,530	G	T	Gln1174His	23 (57)
		2,415,666	G	A	Gly1220Ser	11 (60)
		2,416,390	C	T	Ala1461Val	1 (63)
		2,416,408	T	C	Val1467Ala	31 (41)
		2,416,420	T	C	Leu1471Pro	12 (62)
		2,416,438	T	C	Leu1477Pro	2 (63)
Multidrug resistance-associated protein 2 (PVX_124085)	14	2,043,859	G	C	Gln1407Glu	11 (13)
		2,045,050	C	T	Val1010Met	14 (15)
		2,047,090	G	A	Pro330Ser	1 (15)
		2,047,233	C	A	Arg282Met	13 (14)
		2,047,816	C	G	Glu88Gln	3 (18)
*dhps* (PVX_123230)	14	1,257,856	G	C	Ala383Gly	9 (15)
		1,258,389	C	T	Met205Ile	10 (12)

*Samples column indicates the number of samples with the alternate allele, with the total number of samples genotyped in parentheses

Although chloroquine resistance is common in *P*. *falciparum* in Peru, there is no current evidence of resistance in *P*. *vivax*, and thus, chloroquine remains the first-line treatment for infection [41]. We found several intronic changes in *pvcrt-0* (PVX_087980), which encodes the chloroquine resistance transporter. These changes have previously been detected in a WGS study of *P*. *vivax* in Peru [40]; however, it is not currently well understood what functional changes these SNPs impart. We detected four missense SNPs in *pvmdr1* (PVX_080100), which encodes the multidrug resistance-associated protein 1. Nonsynonymous SNPs in this gene have been associated with chloroquine resistance in prior in vitro assays, in particular a Y976F mutation [42]. While the T958M, M908L, and V221L mutations have been previously detected in Peru and other countries in South America [40, 43, 44], we report the F1070L mutation for the first time in Peru. This mutation is postulated to be a prerequisite for the subsequent acquisition of the Y976F [42] mutation in a two-step mutational path that leads to chloroquine resistance [45]. It has previously been detected in other regions of the world, including Thailand, Indonesia, Turkey, French Guyana, and Azerbaijan [45]. Other *P*. *falciparum* drug resistance-associated gene orthologs with nonsynonymous mutations were *pvdhfr* (PVX_089950), which encodes the bifunctional dihydrofolate reductase-thymidylate synthase enzyme, and *dhps* (PVX_123230), which encodes the dihydropteroate synthetase enzyme. The role of these mutations in phenotypic drug resistance in *P*. *vivax* requires further exploration.

Among these genes, those encoding multidrug resistance protein 2 (PVX_118100) and the multidrug resistance-associated protein 2 (PVX_124085) stood out as having the highest number of SNPs. While the precise function of these genes in *P*. *vivax* has not been well studied, in *P*. *falciparum*, the multidrug resistance-associated protein 2 is considered the most diverse ABC transporter with a potential role in antimalarial resistance and liver stage development [46, 47]. This gene has previously been found to have a high frequency of SNPs in *P*. *falciparum* isolates from Thailand and is thought to modulate the parasite’s response to quinolone antimalarials, which include chloroquine [48].

### Population structure and diversity

We expected that this *P*. *vivax* population would demonstrate high genetic similarity, and consist primarily of monoclonal infections, particularly in the villages of Padre Cocha and Santa Clara which are more remote, consistent with prior studies of *P*. *vivax* in this region [49, 50]. We used rates of heterozygous calls [11] and the *F*_ws_ statistic [28], which calculates the within-host heterozygosity, to determine infection clonality. The majority of the samples were monoclonal (97%), with only two samples (PQSC-105-32 and PQPC-018-0) considered multiclonal based on *F*_ws_ ≤ 0.95 and rate of heterozygous calls > 2× median (Additional file 5: Table S4).

**Fig. 2:**
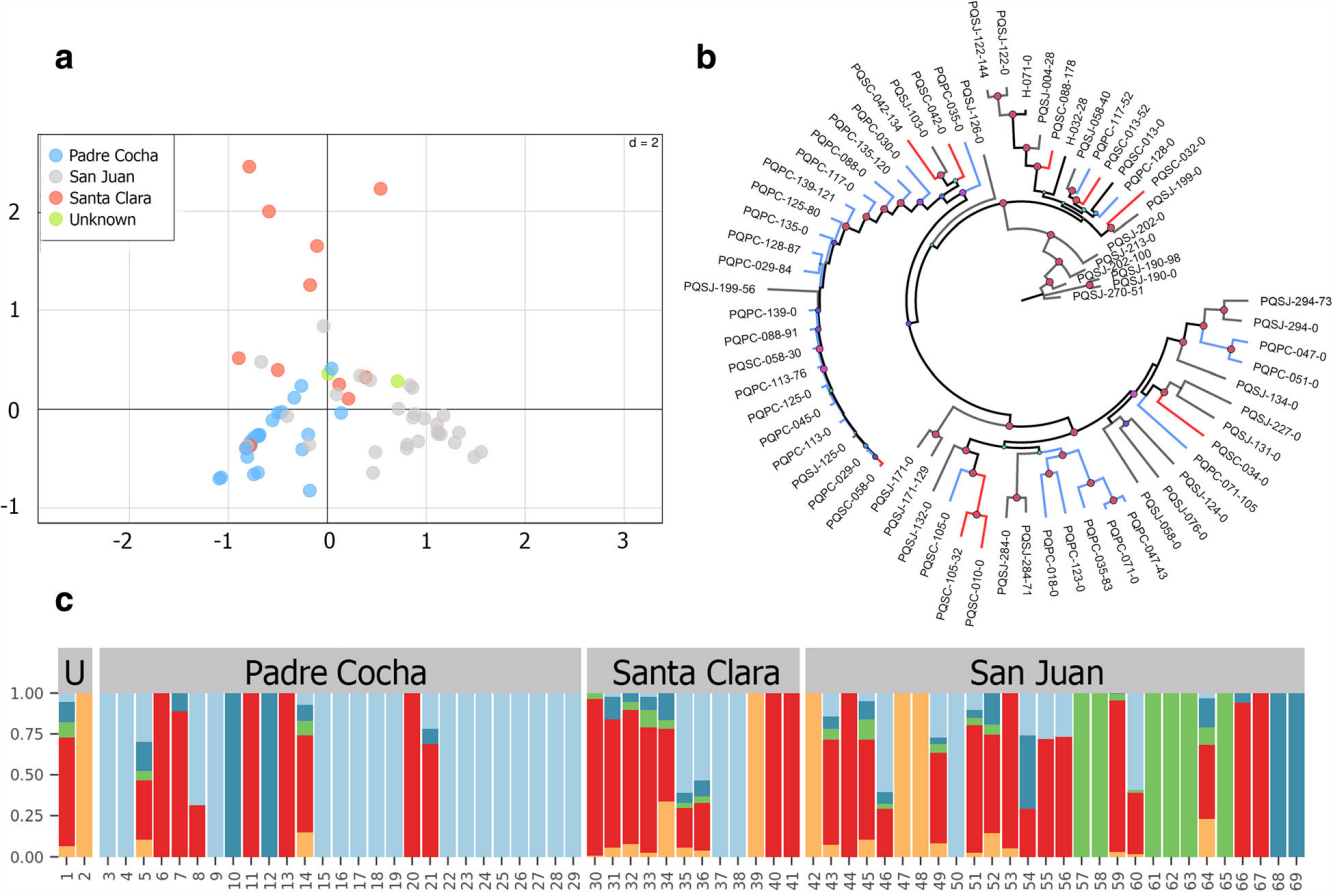
Population structure of *P*. *vivax* samples. **a** Direct analysis of principle components (DAPC) using SNP data from all isolates. Each isolate is colored according to its origin. **b** Unrooted maximum likelihood phylogenetic tree with 1000 bootstraps. Colors correspond to the geographical origin as depicted in **a**. Highlighted red internal circles represent nodes with 100% bootstrap support. **c** Admixture analysis of the variation of the data using 2000 bootstraps. Ancestry for each isolate was established according to a cluster value of 5. Color bars denote the admixture proportion as shown by the *y* axis whereas the *x* axis denotes each of the 69 samples

We used discriminant analysis of principal components (DAPC) on the core SNPs to explore the population structure of *P*. *vivax* from the three sites. DAPC showed that the parasite population in this area is very diverse with some genetic differentiation according to collection site. In this regard, the community of Santa Clara appears to hold the most divergent *P*. *vivax* strains in this sample set (Fig. 2a). The maximum likelihood phylogenetic analysis yielded a tree that was concordant with the DAPC results, emphasizing the high diversity of the parasite population and lack of geographical clustering (Fig. 2b). Given that parasites undergo sexual recombination within the mosquito, this lack of geographical clustering is indicative of gene flow between parasites from these villages. This is consistent with the frequent travel that occurs between the main city of Iquitos and the surrounding villages.

We next performed an ADMIXTURE analysis to assess the maximum likelihood estimation of individual ancestries in this population (Fig. 2c). Similar to the previous analyses of *P*. *vivax* in this region, there was evidence of a highly heterogeneous ancestry among the isolates with sampled genotypes derived from five ancestral populations.

Analysis of the resulting clusters showed that genotypes did not correlate with the geographical location where samples were collected, which could be a result of human population movements across the study sites. This becomes evident by inspecting the site of San Juan, which is located in Iquitos city and is the most important trading center in the Peruvian Amazon. The parasite population of this site contained strains from all five different clusters including isolates with mixed genotypes that share characteristics of all these different populations.

On the contrary, Padre Cocha and Santa Clara, which are located 30 min by river from Iquitos, comprised only three out of the five clusters. These findings contrast with prior studies of *P*. *vivax* in the region, which show high inbreeding and a more clonal population structure [49, 51]. However, results of this study cannot be directly compared to these prior analyses since they were limited by the use of microsatellite data and most of their study sites were located in rural regions with different human migration patterns. This stresses the different epidemiological features of *P*. *vivax* in urban versus rural areas, where higher rates of migration in villages in closer proximity to a large city likely contribute to greater parasite heterogeneity.

### Paired sample analysis

Comparisons between samples obtained from the same subject at the time of initial infection and at the time of recurrent infection revealed an overall high similarity between all isolates with a mean number of 489 segregating sites (Table 2). We used a hidden Markov model to determine regions of the genome that are identical by descent (IBD) [35]. We defined homologous relapses as having segregating sites equal to the mean number of segregating sites overall minus 1.5 standard deviations (segregating sites < 290) and IBD ≥ 99%. This identified a total of 12 homologous relapse pairs. The high similarity of several of the homologous relapse pairs, particularly those from the village of San Juan (PQSJ-122, PQSJ-171, PQSJ-190, PQSJ-284, and PQSJ-294) is reinforced by the maximum likelihood phylogenetic tree topology with bootstrap support values of 100% (Fig. 2b).

**Table 2:** Pairwise comparison between samples obtained from the same subject at the time of initial infection and at the time of recurrent parasitemia

Initial infection	Recurrent parasitemia	Segregating sites	Core genome identical by descent (%)	Microsatellites (concordant/typed)
*PQPC-029-0*	*PQPC-029-84*	*137*	*98.6*	*6/6*
PQPC-035-0	PQPC-035-83	561	4.6	3/6
PQPC-047-0	PQPC-047-43	566	5.6	1/6
PQPC-071-0	PQPC-071-105	560	6.8	1/6
PQPC-088-0	PQPC-088-91	272	98.1	5/6
*PQPC-113-0*	*PQPC-113-76*	*72*	*99.3*	*6/6*
PQPC-117-0	PQPC-117-52	385	2.3	1/4
*PQPC-125-0*	*PQPC-125-80*	*134*	*98.7*	*6/6*
PQPC-128-0	PQPC-128-87	392	98.1	6/6
*PQPC-135-0*	*PQPC-135-120*	*262*	*99.3*	*6/6*
*PQPC-139-0*	*PQPC-139-121*	*111*	*99.3*	*2/3*
PQSC-013-0	PQSC-013-52	337	9.7	2/6
PQSC-042-0	PQSC-042-134	347	52.0	2/6
*PQSC-058-0*	*PQSC-058-30*	*66*	*99.4*	*6/6*
PQSC-105-0	PQSC-105-32	381	25.8	6/6
PQSJ-058-0	PQSJ-058-40	516	8.2	2/6
*PQSJ-122-0*	*PQSJ-122-144*	*61*	*100*	*0/6*
*PQSJ-171-0*	*PQSJ-171-129*	*218*	*99.3*	*6/6*
*PQSJ-190-0*	*PQSJ-190-98*	*117*	*99.7*	*5/6*
PQSJ-199-0	PQSJ-199-56	559	41.2	4/6
*PQSJ-202-0*	*PQSJ-202-100*	*269*	*99.0*	*6/6*
*PQSJ-284-0*	*PQSJ-284-71*	*171*	*99.4*	*6/6*
*PQSJ-294-0*	*PQSJ-294-73*	*200*	*100*	*6/6*

The mean number of segregating sites for all 69 samples included in the study was 489. We identified 12 pairs that are most likely to be homologous relapses, based on the mean number of segregating sites minus 1.5 standard deviations (< 290) and core genome IBD ≥ 99%, which are italicized

**Fig. 3:**
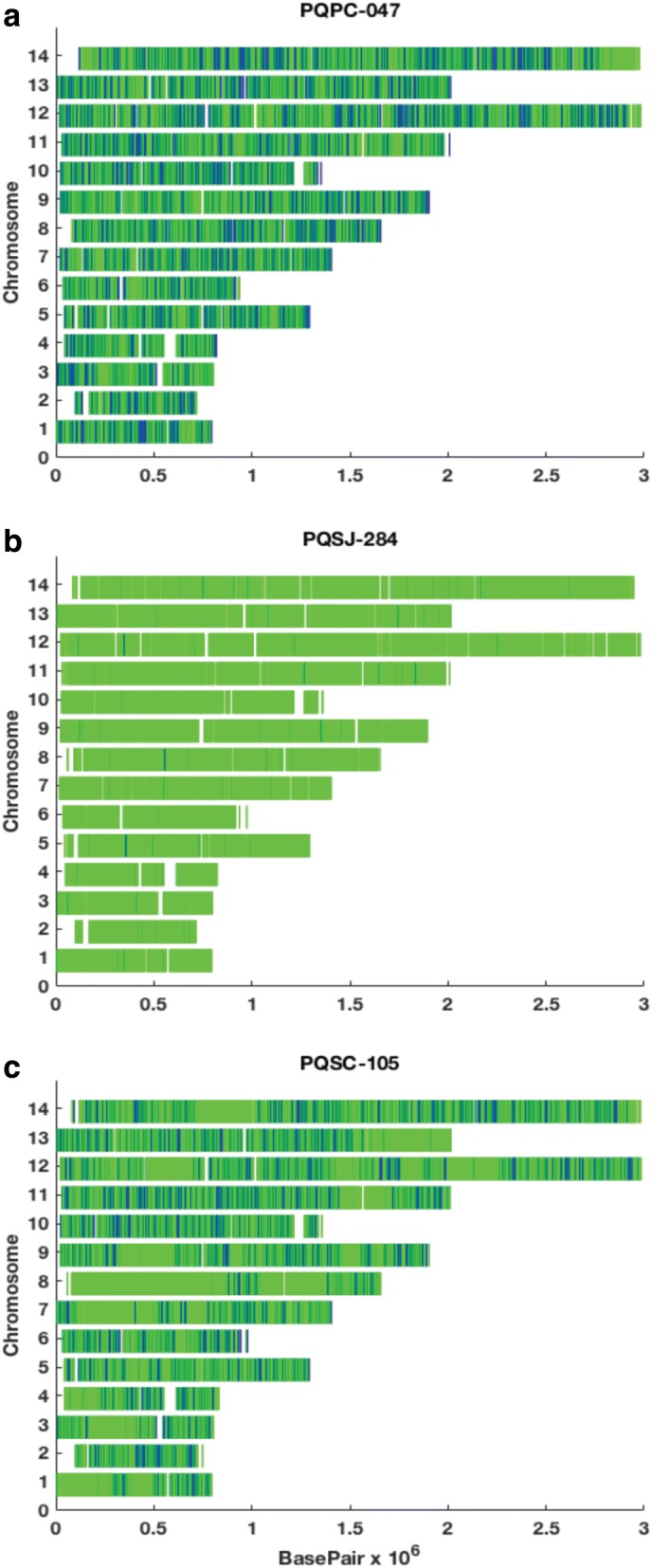
Direct SNP comparisons across the core genome of recurrent infections. Dark blue indicates sites where the paired samples are discordant while green demonstrates where they are concordant. **a** A recurrent infection with a heterologous *P*. *vivax* strain from paired samples from subject PQPC-047 (5.6% identical by descent (IBD)). **b** A homologous relapse from subject PQSJ-284 (99.4% IBD). **c** A possible heterologous relapse with large shared blocks of concordant SNPs from subject PQSC-105 (25.8% IBD)

In addition, we sought to identify potential heterologous relapses caused by *P*. *vivax* meiotic siblings, which can occur due to recombination and outcrossing in the mosquito midgut during the initial infection [9]. We performed direct SNP comparisons across the core genome for all *P*. *vivax* pairs to help differentiate heterologous infections (Fig. 3a) from homologous relapses (Fig. 3b) and to identify highly related pairs, which share long blocks of concordant SNPs (Fig. 3c). These highly related pairs could be meiotic siblings or may represent heterologous relapses that represent reactivation of hypnozoites from the initial infection and another genetically different infection. We identified a total of three potential pairs: PQSC-042, PQSC-105, and PQSJ-199. These sample pairs had 52.0, 25.8, and 41.2% of their genomes that were IBD, in contrast with the homologous relapse pairs that had IBDs greater than 98%.

We compared our results to the microsatellite genotyping that was done during the original study (Table 2). Overall, results were concordant for 17 of the 23 pairs (73.9%). Microsatellite data was concordant for 9 of the 12 homologous relapses we identified with whole genome sequencing. There were two samples that were homologous by microsatellite markers but not by our data (PQPC-128 and PQSC-105). The PQSC-105 pair is one of the highly related pairs described above. Microsatellite markers may have misidentified this pair because the genomic areas genotyped with the markers could have been identical despite differences in the rest of the genome. The PQPC-128 pair had the lowest number of informative sites for both samples on whole genome sequencing and thus may have been misidentified as a heterologous infection by our method. In addition, there were three pairs that were classified as heterologous infections by microsatellites, but were homologous relapses based on our data (PQPC-139, PQSJ-122, and PQSJ-190). The PQPC-139 pair could only be genotyped at three sites, PQSJ-122 had 0/6 markers concordant, and the PQSJ-190 pair was similar at 5/6 markers. This may represent microsatellite errors. Altogether, the comparison demonstrates how microsatellite markers may identify homologous relapses with high specificity, but also can demonstrate lower sensitivity compared to whole genome sequencing.

In the clinical trial, subjects who received the 5-day regimen had a significantly higher rate of homologous relapses, while subjects who received the 7- or 14-day regimens did not have significantly different rates of homologous relapses. From our paired sample comparison, we noted a trend towards a higher rate of relapse in subjects who received a shorter duration of treatment, with a relapse rate of 100% (1/1) with the 5-day regimen, 66.7% (6/9) in the 7-day regimen group, and 41.7% (5/12) with the 14-day regimen, although the sample size was not large enough to reach statistical significance.

The presence of these homologous relapses underscored the need to assess the host genetics to identify if they occurred due to alterations in primaquine metabolism. It is known that the human CYP2D6 enzyme, encoded by the highly polymorphic *cyp2d6* gene, is important in the metabolism of many drugs, including primaquine. In this regard, poor or intermediate activity CYP2D6 phenotypes have been associated with an increased risk for *P*. *vivax* relapse following treatment with primaquine [52, 53]. CYP2D6 phenotypes were assessed in all homologous relapse pairs in our sample set. Eight of these ten pairs were classified as extensive metabolizers (at least one allele coding for an enzyme with normal activity), and four were classified as intermediate metabolizers (heterozygous for one null and one active allele). Thus, the CYP2D6 poor or intermediate phenotype did not explain the majority of homologous relapses in our study.

We further analyzed each homologous relapse pair to identify SNPs that emerged after treatment but were not present in the initial infection to identify genetic changes that arose as a result of drug or immune system pressures. Three homologous relapses had missense mutations found in a sporozoite and liver-stage asparagine-rich protein (PVX_092945) that were not present in the initial infection: PQPC-029 (N647I, A646T), PQPC-125 (N647I, A646T), and PQSJ-171 (A654G). The protein encoded by this gene is specifically expressed in sporozoites and during liver stage development and may function as a regulator of gene expression during liver stage replication [54]. We identified two heterozygous mutations in multidrug resistance protein 2 (PVX_118100) in the relapse sample for PQSJ-122 (V1467A, L1471P) which were not present in the initial infection.

## Discussion

This study provides an extensive dataset of population genetics of *Plasmodium vivax* in the Peruvian Amazon. We define the parasite population structure in this region using whole genome sequencing and highlight a novel method for distinguishing homologous relapses from reinfections or heterologous relapses. To the best of our knowledge, this is the first large-scale study to evaluate recurrent *P*. *vivax* infections using whole genome sequencing.

Our paired sample analysis and comparison to prior microsatellite genotyping demonstrate that whole genome sequencing has increased sensitivity for detecting homologous relapses and relapses due to highly related meiotic siblings. Upon comparison of our data to the prior microsatellite genotyping, we found that microsatellites failed to detect some homologous relapses. In addition, microsatellites misidentified a highly related pair that likely represented a heterologous relapse with meiotic siblings. We cannot definitively determine how using our method could have changed the outcomes of the clinical trial without performing analysis on a larger number of samples. However, using whole genome sequencing likely would lead to the identification of an increased number of homologous relapses and thus possible primaquine failures. While the original study did not find a significant difference between the 0.5 mg/kg × 7 days and 0.25 mg/kg × 14 days primaquine dosing regimens, we did note a trend in our data towards a higher rate of relapse with the shorter duration of treatment. Thus, the method of comparing recurrent *P*. *vivax infections* implemented in our study would improve the assessment of antirelapse therapy efficacy during clinical trials performed in endemic settings. Further evaluation should be done using this method in future clinical trials of known and novel antirelapse therapies, especially as the cost of whole genome sequencing continues to decline and new methods such as SWGA are developed to enrich *P*. *vivax* DNA directly from subject samples.

We identified several mutations in many genes orthologous to multidrug resistance genes in *P. falciparum*, with a particularly high SNP rate in *pvmrp2*, and several novel alleles noted in others, although it remains unclear at this time what functional changes these impart. Although none of the resulting genotypes have been associated with resistance in *P*. *vivax*, the high diversity in drug resistance genes in the core genome underscores the potential risk for the emergence and spread of resistance. These findings highlight how little is known about the genetic basis of drug resistance in *P*. *vivax*. One major reason is the lack of a robust in vitro culture system for *P*. *vivax* compared to *P*. *falciparum*. In this study, we demonstrate that SWGA is a useful tool for enriching the amount of *P*. *vivax* DNA in unprocessed samples to improve the efficiency of WGS.

Our assessment of the population structure of *P*. *vivax* parasites in this region reveals a high level of diversity with evidence of recombination among isolates across these villages. In addition, genetic clustering by DAPC suggested very little differentiation according to the sampling sites. This finding was also confirmed by genetic clustering using ADMIXTURE that revealed at least five parasite clusters on our population with no separation by geographic location. The low level of differentiation among parasites according to site could be due to high mobilization of people between villages and within the city of Iquitos. It is important to note that a series of campaigns aimed at preventing and controlling malaria were executed during the period of sample collection. These activities were funded under the Global Fund initiative to control malaria in the border areas of the Andean region (PAMAFRO project). These campaigns were successful at lowering malaria incidence up to nearly 50% until 2011 when the project ended [41]. Therefore, it is possible that reduced gene flow and diversity could be a result of the impact of the intervention on the parasite population in the region. Further studies are needed to evaluate the dynamics of the parasite population and the effects of this major intervention on the evolution of malaria in this setting, especially given the sustained increase in malaria rates after PAMAFRO.

Our study had several limitations. Performing whole genome sequencing on *P*. *vivax* directly from subject samples currently remains costly and inefficient without enrichment techniques such as SWGA. It is important to obtain high-quality whole genome sequences to perform paired sample comparisons since one of the pairs with a low number of informative sites may have been misclassified as a heterologous infection. However, due to uneven amplification across the genome with SWGA, it is more difficult to detect copy number variants, and thus, we were not able to perform this analysis. In addition, SWGA may amplify the majority clone in a multiclonal sample, thus potentially increasing the number of monoclonal samples [19]. Our finding of a majority of monoclonal samples, with only one multiclonal sample in Santa Clara and one in Padre Cocha, was not entirely consistent with other studies of this region in the Peruvian Amazon. Since the population of San Juan has the highest mobility, it would be expected that multiclonal samples would be more common at that site.

In addition, despite the high sensitivity of whole genome sequencing, it remains challenging to distinguish reinfections from relapses in a malaria-endemic area with genetically similar *P*. *vivax* isolates. It also remains impossible to distinguish a heterologous relapse from reinfection without being able to genotype all the hypnozoites that a person carries in their liver. However, we identified homologous relapses based on pairwise similarity compared to the similarity of the entire population and utilized a stringent cutoff. Finally, due to small sample size, we were unable to perform a genome-wide association study to identify SNPs associated with relapse. We were thus unable to identify particular SNPs that were associated with homologous relapses and thus could be implicated as an underlying genetic mechanism of primaquine resistance.

## Conclusions

Overall, our study shows that whole genome sequencing is a highly sensitive tool for gathering information about potential drug resistance, identifying homologous relapses with improved accuracy, and analyzing population structure and gene flow, especially as the cost of this technology continues to decline rapidly. Despite the significant reduction of malaria prevalence worldwide, the changing epidemiology of *P*. *vivax* malaria due to the presence of asymptomatic infections that can still transmit the disease and the risk of relapses challenges sustainable progress towards its elimination. These limitations require research that can help us to elucidate the changing landscape of *P*. *vivax* transmission, better understand the genetic diversity of *P*. *vivax* and allow us to monitor the efficacy of antirelapse treatment.

## Additional files

Additional file 1: **Table S1.** Study information for 46 of the *P. vivax* whole genome sequences obtained from 3 villages surrounding Iquitos, Peru. Data for 23 subjects was unavailable. (XLSX 11 kb)

Additional file 2: **Table S2.** Sequencing statistics for 69 *Plasmodium vivax* sequences collected in 3 villages in the Peruvian Amazon after alignment to the *P. vivax* reference genome. (XLSX 13 kb)

Additional file 3: **Table S3.** High quality single nucleotide polymorphisms in the core genome of the 69 *P. vivax* sequences. Samples are listed starting at column M and are labeled with the subject code followed by the day of collection. (XLSX 17419 kb)

Additional file 4: **Figure S1.** Genome wide SNP density. Mean number of single nucleotide polymorphisms (SNPs) across each of the 14 *Plasmodium vivax* chromosomes. SNP dense regions were identified in chromosomes 3, 6, 10 and 13. (TIF 3188 kb)

Additional file 5: **Table S4.** Clonality calculations for the 69 *P. vivax* samples using the Fws (within-host heterozygosity) statistic and number of heterozygous calls in the core single nucleotide polymorphisms (SNPs). As expected, the majority of samples were monoclonal (97%) and only two samples (highlighted) were multiclonal based on Fws ≤ 0.95 and number of heterozygous calls greater than two times the median. (XLSX 11 kb)

## Abbreviations

CYP2D6: Cytochrome P450 2D6
DAPC: Discriminant analysis of principal components
IBD: Identical by descent
SNP: Single nucleotide polymorphism
SWGA: Selective whole genome amplification
