# Supplementary figures and images for: Exploration of Plasmodium vivax transmission dynamics and recurrent infections in the Peruvian Amazon using whole genome sequencing
Source: Genome Med. 2018 Jul 4;10:52. doi: 10.1186/s13073-018-0563-0 (PMC6032790; doi:10.1186/s13073-018-0563-0)

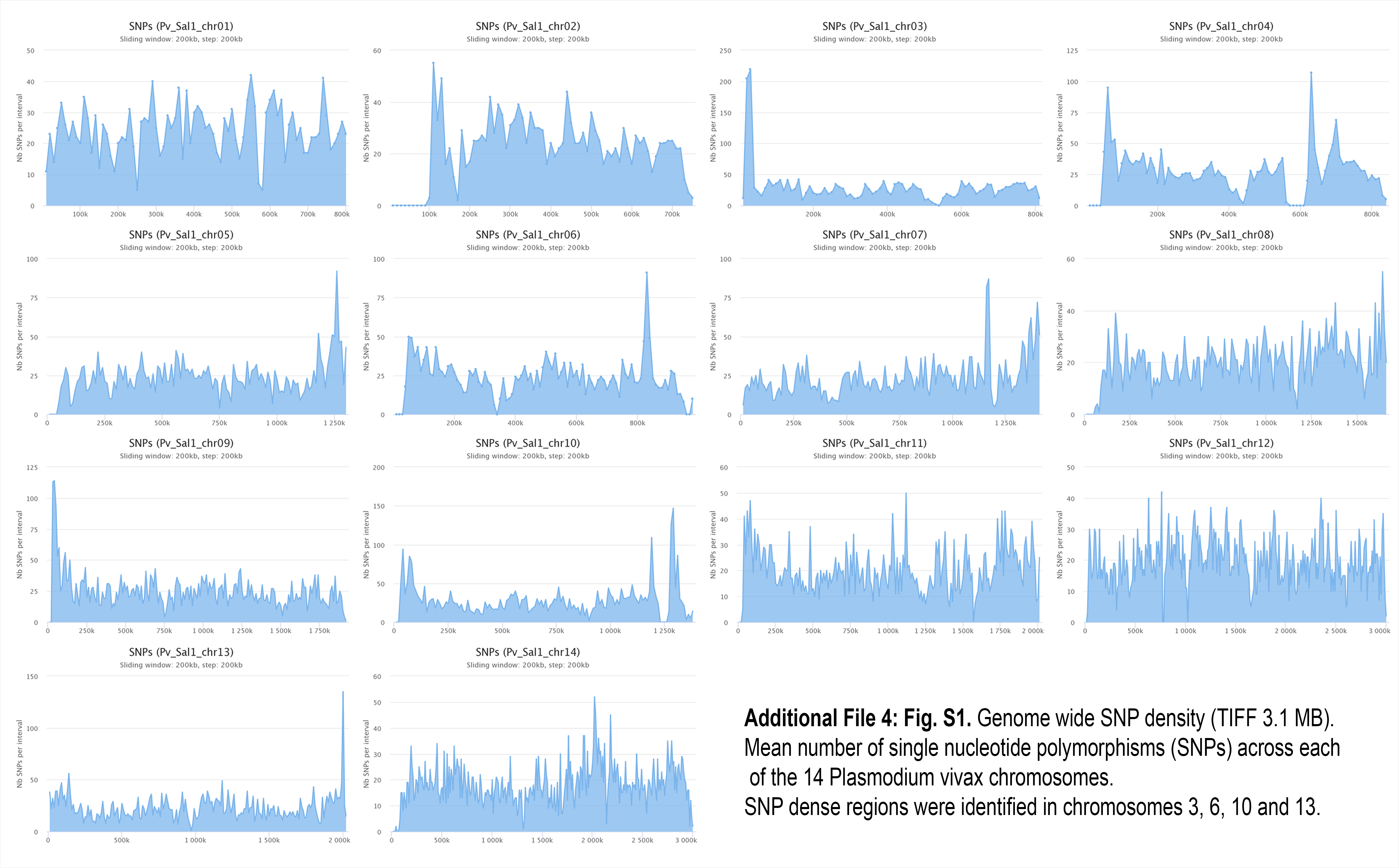

Supplement: Supplementary file 4 — Figure S1. Genome wide SNP density. Mean number of single nucleotide polymorphisms (SNPs) across each of the 14 Plasmodium vivax chromosomes. SNP dense regions were identified in chromosomes 3, 6, 10 and 13. (TIF 3188 kb) [file 13073_2018_563_MOESM4_ESM.tif]
